# A new framework for developing and evaluating complex interventions: update of Medical Research Council guidance

**DOI:** 10.1136/bmj.n2061

**Published:** 2021-09-30

**Authors:** Kathryn Skivington, Lynsay Matthews, Sharon Anne Simpson, Peter Craig, Janis Baird, Jane M Blazeby, Kathleen Anne Boyd, Neil Craig, David P French, Emma McIntosh, Mark Petticrew, Jo Rycroft-Malone, Martin White, Laurence Moore

**Affiliations:** 1 MRC/CSO Social and Public Health Sciences Unit, Institute of Health and Wellbeing, University of Glasgow, Glasgow, UK; 2 Medical Research Council Lifecourse Epidemiology Unit, University of Southampton, Southampton, UK; 3 Medical Research Council ConDuCT-II Hub for Trials Methodology Research and Bristol Biomedical Research Centre, Bristol, UK; 4 Health Economics and Health Technology Assessment Unit, Institute of Health and Wellbeing, University of Glasgow, Glasgow, UK; 5 Public Health Scotland, Glasgow, UK; 6 Manchester Centre for Health Psychology, University of Manchester, Manchester, UK; 7 London School of Hygiene and Tropical Medicine, London, UK; 8 Faculty of Health and Medicine, Lancaster University, Lancaster, UK; 9 Medical Research Council Epidemiology Unit, University of Cambridge, Cambridge, UK

## Abstract

The UK Medical Research Council’s widely used guidance for developing and evaluating complex interventions has been replaced by a new framework, commissioned jointly by the Medical Research Council and the National Institute for Health Research, which takes account of recent developments in theory and methods and the need to maximise the efficiency, use, and impact of research.

Complex interventions are commonly used in the health and social care services, public health practice, and other areas of social and economic policy that have consequences for health. Such interventions are delivered and evaluated at different levels, from individual to societal levels. Examples include a new surgical procedure, the redesign of a healthcare programme, and a change in welfare policy. The UK Medical Research Council (MRC) published a framework for researchers and research funders on developing and evaluating complex interventions in 2000 and revised guidance in 2006.^1^ ^2^ ^3^ Although these documents continue to be widely used and are now accompanied by a range of more detailed guidance on specific aspects of the research process,^4^ ^5^ ^6^ ^7^ ^8^ several important conceptual, methodological and theoretical developments have taken place since 2006. These developments have been included in a new framework commissioned by the National Institute of Health Research (NIHR) and the MRC.^9^ The framework aims to help researchers work with other stakeholders to identify the key questions about complex interventions, and to design and conduct research with a diversity of perspectives and appropriate choice of methods.

**Summary points**

Complex intervention research can take an efficacy, effectiveness, theory based, and/or systems perspective, the choice of which is based on what is known already and what further evidence would add most to knowledge
Complex intervention research goes beyond asking whether an intervention works in the sense of achieving its intended outcome—to asking a broader range of questions (eg, identifying what other impact it has, assessing its value relative to the resources required to deliver it, theorising how it works, taking account of how it interacts with the context in which it is implemented, how it contributes to system change, and how the evidence can be used to support real world decision making)
A trade-off exists between precise unbiased answers to narrow questions and more uncertain answers to broader, more complex questions; researchers should answer the questions that are most useful to decision makers rather than those that can be answered with greater certainty
Complex intervention research can be considered in terms of phases, although these phases are not necessarily sequential: development or identification of an intervention, assessment of feasibility of the intervention and evaluation design, evaluation of the intervention, and impactful implementation
At each phase, six core elements should be considered to answer the following questions:
  - How does the intervention interact with its context?
  - What is the underpinning programme theory?
  - How can diverse stakeholder perspectives be included in the research?
  - What are the key uncertainties?
  - How can the intervention be refined?
  - What are the comparative resource and outcome consequences of the intervention?
The answers to these questions should be used to decide whether the research should proceed to the next phase, return to a previous phase, repeat a phase, or stop

## Development of the Framework for Developing and Evaluating Complex Interventions

The updated Framework for Developing and Evaluating Complex Interventions is the culmination of a process that included four stages:

- A gap analysis to identify developments in the methods and practice since the previous framework was published
- A full day expert workshop, in May 2018, of 36 participants to discuss the topics identified in the gap analysis
- An open consultation on a draft of the framework in April 2019, whereby we sought stakeholder opinion by advertising via social media, email lists and other networks for written feedback (52 detailed responses were received from stakeholders internationally)
- Redraft using findings from the previous stages, followed by a final expert review.

We also sought stakeholder views at various interactive workshops throughout the development of the framework: at the annual meetings of the Society for Social Medicine and Population Health (2018), the UK Society for Behavioural Medicine (2017, 2018), and internationally at the International Congress of Behavioural Medicine (2018). The entire process was overseen by a scientific advisory group representing the range of relevant NIHR programmes and MRC population health investments. The framework was reviewed by the MRC-NIHR Methodology Research Programme Advisory Group and then approved by the MRC Population Health Sciences Group in March 2020 before undergoing further external peer and editorial review through the NIHR Journals Library peer review process. More detailed information and the methods used to develop this new framework are described elsewhere.^9^ This article introduces the framework and summarises the main messages for producers and users of evidence.

## What are complex interventions?

An intervention might be considered complex because of properties of the intervention itself, such as the number of components involved; the range of behaviours targeted; expertise and skills required by those delivering and receiving the intervention; the number of groups, settings, or levels targeted; or the permitted level of flexibility of the intervention or its components. For example, the Links Worker Programme was an intervention in primary care in Glasgow, Scotland, that aimed to link people with community resources to help them “live well” in their communities. It targeted individual, primary care (general practitioner (GP) surgery), and community levels. The intervention was flexible in that it could differ between primary care GP surgeries. In addition, the Link Workers did not support just one specific health or wellbeing issue: bereavement, substance use, employment, and learning difficulties were all included.^10^ ^11^ The complexity of this intervention had implications for many aspects of its evaluation, such as the choice of appropriate outcomes and processes to assess.

Flexibility in intervention delivery and adherence might be permitted to allow for variation in how, where, and by whom interventions are delivered and received. Standardisation of interventions could relate more to the underlying process and functions of the intervention than on the specific form of components delivered.^12^ For example, in surgical trials, protocols can be designed with flexibility for intervention delivery.^13^ Interventions require a theoretical deconstruction into components and then agreement about permissible and prohibited variation in the delivery of those components. This approach allows implementation of a complex intervention to vary across different contexts yet maintain the integrity of the core intervention components. Drawing on this approach in the ROMIO pilot trial, core components of minimally invasive oesophagectomy were agreed and subsequently monitored during main trial delivery using photography.^14^

Complexity might also arise through interactions between the intervention and its context, by which we mean “any feature of the circumstances in which an intervention is conceived, developed, implemented and evaluated.”^6^ ^15^ ^16^ ^17^ Much of the criticism of and extensions to the existing framework and guidance have focused on the need for greater attention on understanding how and under what circumstances interventions bring about change.^7^ ^15^ ^18^ The importance of interactions between the intervention and its context emphasises the value of identifying mechanisms of change, where mechanisms are the causal links between intervention components and outcomes; and contextual factors, which determine and shape whether and how outcomes are generated.^19^

Thus, attention is given not only to the design of the intervention itself but also to the conditions needed to realise its mechanisms of change and/or the resources required to support intervention reach and impact in real world implementation. For example, in a cluster randomised trial of ASSIST (a peer led, smoking prevention intervention), researchers found that the intervention worked particularly well in cohesive communities that were served by one secondary school where peer supporters were in regular contact with their peers—a key contextual factor consistent with diffusion of innovation theory, which underpinned the intervention design.^20^ A process evaluation conducted alongside a trial of robot assisted surgery identified key contextual factors to support effective implementation of this procedure, including engaging staff at different levels and surgeons who would not be using robot assisted surgery, whole team training, and an operating theatre of suitable size.^21^

With this framing, complex interventions can helpfully be considered as events in systems.^16^ Thinking about systems helps us understand the interaction between an intervention and the context in which it is implemented in a dynamic way.^22^ Systems can be thought of as complex and adaptive,^23^ characterised by properties such as emergence, feedback, adaptation, and self-organisation (table 1).

**Table 1:** Properties and examples of complex adaptive systems

System properties	Example
**Emergence**	
Complex systems have emergent, often unanticipated, properties that are a feature of the system as a whole	Group based interventions that target young people at risk could be undermined by the emergence of new social relationships among the group that increase members’ exposure to risk behaviours, while reducing their contact with other young people less tolerant of risk taking^24^
**Feedback**	
Where one change reinforces, promotes, balances, or diminishes another	A smoking ban in public places reduces the visibility and convenience of smoking; fewer young people start smoking, further reducing its visibility, in a reinforcing loop^22^
**Adaptation**	
Change of system behaviour in response to an intervention	Retailers adapted to the ban on multi-buy discounts by discounting individual alcohol products, offering them at the same price individually as they would have been if part of a multi-buy offer^25^
**Self-organisation**	
Order arising from spontaneous local interaction rather than a preconceived plan or external control	Recognising that individual treatment did not address some social aspects of alcohol dependency, recovering drinkers self-organised to form Alcoholics Anonymous

For complex intervention research to be most useful to decision makers, it should take into account the complexity that arises both from the intervention’s components and from its interaction with the context in which it is being implemented.

## Research perspectives

The previous framework and guidance were based on a paradigm in which the salient question was to identify whether an intervention was effective. Complex intervention research driven primarily by this question could fail to deliver interventions that are implementable, cost effective, transferable, and scalable in real world conditions. To deliver solutions for real world practice, complex intervention research requires strong and early engagement with patients, practitioners, and policy makers, shifting the focus from the “binary question of effectiveness”^26^ to whether and how the intervention will be acceptable, implementable, cost effective, scalable, and transferable across contexts. In line with a broader conception of complexity, the scope of complex intervention research needs to include the development, identification, and evaluation of whole system interventions and the assessment of how interventions contribute to system change.^22^ ^27^ The new framework therefore takes a pluralistic approach and identifies four perspectives that can be used to guide the design and conduct of complex intervention research: efficacy, effectiveness, theory based, and systems (table 2).

**Table 2:** Research perspectives

Perspective and research question	Key points	Vaccine study example
**Efficacy**		
To what extent does the intervention produce the intended outcomes in experimental or ideal settings?	Conducted under idealised conditions; maximises internal validity to provide a precise, unbiased estimate of efficacy	Seeks to measure the effect of the vaccine on immune system response and report its safety^28^
**Effectiveness**		
To what extent does the intervention produce the intended outcomes in real world settings?	Intervention often compared against treatment as usual; results inform choices between an established and a novel approach to achieving the desired outcome	Seeks to determine whether the vaccination programme, implemented in a range of real world populations and settings, is effective in terms of what it set out to do (eg, prevent disease)^29^
**Theory based**		
What works in which circumstances and how?	Aims to understand how change is brought about, including the interplay of mechanisms and context; can lead to refinement of theory	Asks why effectiveness varies across contexts, and asks what this variation indicates about the conditions for a successful vaccination programme^30^; considerations that might be explored go beyond whether the vaccine works^31^
**Systems**		
How do the system and intervention adapt to one another?	Treats the intervention as a disruption to a complex system^16^	Seeks to understand the dynamic interdependence of vaccination rollout, population risk of infection and willingness to be vaccinated, as the vaccination programme proceeds^32^

Although each research perspective prompts different types of research question, they should be thought of as overlapping rather than mutually exclusive. For example, theory based and systems perspectives to evaluation can be used in conjunction,^33^ while an effectiveness evaluation can draw on a theory based or systems perspective through an embedded process evaluation to explore how and under what circumstances outcomes are achieved.^34^ ^35^ ^36^

Most complex health intervention research so far has taken an efficacy or effectiveness perspective and for some research questions these perspectives will continue to be the most appropriate. However, some questions equally relevant to the needs of decision makers cannot be answered by research restricted to an efficacy or effectiveness perspective. A wider range and combination of research perspectives and methods, which answer questions beyond efficacy and effectiveness, need to be used by researchers and supported by funders. Doing so will help to improve the extent to which key questions for decision makers can be answered by complex intervention research. Example questions include:

- Will this effective intervention reproduce the effects found in the trial when implemented here?
- Is the intervention cost effective?
- What are the most important things we need to do that will collectively improve health outcomes?
- In the absence of evidence from randomised trials and the infeasibility of conducting such a trial, what does the existing evidence suggest is the best option now and how can this be evaluated?
- What wider changes will occur as a result of this intervention?
- How are the intervention effects mediated by different settings and contexts?

## Phases and core elements of complex intervention research

**Fig 1:**
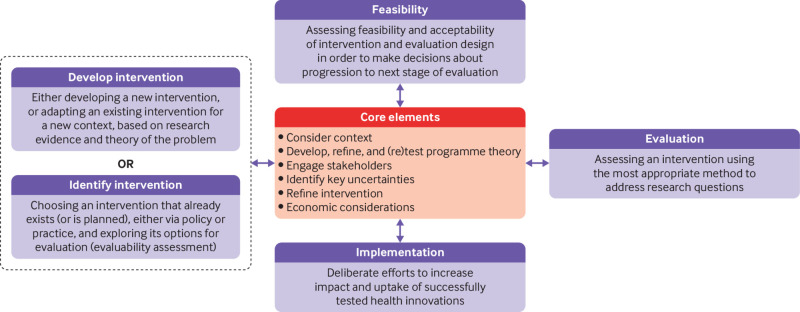
Framework for developing and evaluating complex interventions. Context=any feature of the circumstances in which an intervention is conceived, developed, evaluated, and implemented; programme theory=describes how an intervention is expected to lead to its effects and under what conditions—the programme theory should be tested and refined at all stages and used to guide the identification of uncertainties and research questions; stakeholders=those who are targeted by the intervention or policy, involved in its development or delivery, or more broadly those whose personal or professional interests are affected (that is, who have a stake in the topic)—this includes patients and members of the public as well as those linked in a professional capacity; uncertainties=identifying the key uncertainties that exist, given what is already known and what the programme theory, research team, and stakeholders identify as being most important to discover—these judgments inform the framing of research questions, which in turn govern the choice of research perspective; refinement=the process of fine tuning or making changes to the intervention once a preliminary version (prototype) has been developed; economic considerations=determining the comparative resource and outcome consequences of the interventions for those people and organisations affected

The framework divides complex intervention research into four phases: development or identification of the intervention, feasibility, evaluation, and implementation (fig 1). A research programme might begin at any phase, depending on the key uncertainties about the intervention in question. Repeating phases is preferable to automatic progression if uncertainties remain unresolved. Each phase has a common set of core elements—considering context, developing and refining programme theory, engaging stakeholders, identifying key uncertainties, refining the intervention, and economic considerations. These elements should be considered early and continually revisited throughout the research process, and especially before moving between phases (for example, between feasibility testing and evaluation).

### Core elements

#### Context

The effects of a complex intervention might often be highly dependent on context, such that an intervention that is effective in some settings could be ineffective or even harmful elsewhere.^6^ As the examples in table 1 show, interventions can modify the contexts in which they are implemented, by eliciting responses from other agents, or by changing behavioural norms or exposure to risk, so that their effects will also vary over time. Context can be considered as both dynamic and multi-dimensional. Key dimensions include physical, spatial, organisational, social, cultural, political, or economic features of the healthcare, health system, or public health contexts in which interventions are implemented. For example, the evaluation of the Breastfeeding In Groups intervention found that the context of the different localities (eg, staff morale and suitable premises) influenced policy implementation and was an explanatory factor in why breastfeeding rates increased in some intervention localities and declined in others.^37^

#### Programme theory

Programme theory describes how an intervention is expected to lead to its effects and under what conditions. It articulates the key components of the intervention and how they interact, the mechanisms of the intervention, the features of the context that are expected to influence those mechanisms, and how those mechanisms might influence the context.^38^ Programme theory can be used to promote shared understanding of the intervention among diverse stakeholders, and to identify key uncertainties and research questions. Where an intervention (such as a policy) is developed by others, researchers still need to theorise the intervention before attempting to evaluate it.^39^ Best practice is to develop programme theory at the beginning of the research project with involvement of diverse stakeholders, based on evidence and theory from relevant fields, and to refine it during successive phases. The EPOCH trial tested a large scale quality improvement programme aimed at improving 90 day survival rates for patients undergoing emergency abdominal surgery; it included a well articulated programme theory at the outset, which supported the tailoring of programme delivery to local contexts.^40^ The development, implementation, and post-study reflection of the programme theory resulted in suggested improvements for future implementation of the quality improvement programme.

A refined programme theory is an important evaluation outcome and is the principal aim where a theory based perspective is taken. Improved programme theory will help inform transferability of interventions across settings and help produce evidence and understanding that is useful to decision makers. In addition to full articulation of programme theory, it can help provide visual representations—for example, using a logic model,^41^ ^42^ ^43^ realist matrix,^44^ or a system map,^45^ with the choice depending on which is most appropriate for the research perspective and research questions. Although useful, any single visual representation is unlikely to sufficiently articulate the programme theory—it should always be articulated well within the text of publications, reports, and funding applications.

#### Stakeholders

Stakeholders include those individuals who are targeted by the intervention or policy, those involved in its development or delivery, or those whose personal or professional interests are affected (that is, all those who have a stake in the topic). Patients and the public are key stakeholders. Meaningful engagement with appropriate stakeholders at each phase of the research is needed to maximise the potential of developing or identifying an intervention that is likely to have positive impacts on health and to enhance prospects of achieving changes in policy or practice. For example, patient and public involvement^46^ activities in the PARADES programme, which evaluated approaches to reduce harm and improve outcomes for people with bipolar disorder, were wide ranging and central to the project.^47^ Involving service users with lived experiences of bipolar disorder had many benefits, for example, it enhanced the intervention but also improved the evaluation and dissemination methods. Service users involved in the study also had positive outcomes, including more settled employment and progression to further education. Broad thinking and consultation is needed to identify a diverse range of appropriate stakeholders.

The purpose of stakeholder engagement will differ depending on the context and phase of the research, but is essential for prioritising research questions, the co-development of programme theory, choosing the most useful research perspective, and overcoming practical obstacles to evaluation and implementation. Researchers should nevertheless be mindful of conflicts of interest among stakeholders and use transparent methods to record potential conflicts of interest. Research should not only elicit stakeholder priorities, but also consider why they are priorities. Careful consideration of the appropriateness and methods of identification and engagement of stakeholders is needed.^46^ ^48^

#### Key uncertainties

Many questions could be answered at each phase of the research process. The design and conduct of research need to engage pragmatically with the multiple uncertainties involved and offer a flexible and emergent approach to exploring them.^15^ Therefore, researchers should spend time developing the programme theory, clearly identifying the remaining uncertainties, given what is already known and what the research team and stakeholders identify as being most important to determine. Judgments about the key uncertainties inform the framing of research questions, which in turn govern the choice of research perspective.

Efficacy trials of relatively uncomplicated interventions in tightly controlled conditions, where research questions are answered with great certainty, will always be important, but translation of the evidence into the diverse settings of everyday practice is often highly problematic.^27^ For intervention research in healthcare and public health settings to take on more challenging evaluation questions, greater priority should be given to mixed methods, theory based, or systems evaluation that is sensitive to complexity and that emphasises implementation, context, and system fit. This approach could help improve understanding and identify important implications for decision makers, albeit with caveats, assumptions, and limitations.^22^ Rather than maintaining the established tendency to prioritise strong research designs that answer some questions with certainty but are unsuited to resolving many important evaluation questions, this more inclusive, deliberative process could place greater value on equivocal findings that nevertheless inform important decisions where evidence is sparse.

#### Intervention refinement

Within each phase of complex intervention research and on transition from one phase to another, the intervention might need to be refined, on the basis of data collected or development of programme theory.^4^ The feasibility and acceptability of interventions can be improved by engaging potential intervention users to inform refinements. For example, an online physical activity planner for people with diabetes mellitus was found to be difficult to use, resulting in the tool providing incorrect personalised advice. To improve usability and the advice given, several iterations of the planner were developed on the basis of interviews and observations. This iterative process led to the refined planner demonstrating greater feasibility and accuracy.^49^

Refinements should be guided by the programme theory, with acceptable boundaries agreed and specified at the beginning of each research phase, and with transparent reporting of the rationale for change. Scope for refinement might also be limited by the policy or practice context. Refinement will be rare in the evaluation phase of efficacy and effectiveness research, where interventions will ideally not change or evolve within the course of the study. However, between the phases of research and within systems and theory based evaluation studies, refinement of interventions in response to accumulated data or as an adaptive and variable response to context and system change are likely to be desirable features of the intervention and a key focus of the research.

#### Economic considerations

Economic evaluation—the comparative analysis of alternative courses of action in terms of both costs (resource use) and consequences (outcomes, effects)—should be a core component of all phases of intervention research. Early engagement of economic expertise will help identify the scope of costs and benefits to assess in order to answer questions that matter most to decision makers.^50^ Broad ranging approaches such as cost benefit analysis or cost consequence analysis, which seek to capture the full range of health and non-health costs and benefits across different sectors,^51^ will often be more suitable for an economic evaluation of a complex intervention than narrower approaches such as cost effectiveness or cost utility analysis. For example, evaluation of the New Orleans Intervention Model for infants entering foster care in Glasgow included short and long term economic analysis from multiple perspectives (the UK’s health service and personal social services, public sector, and wider societal perspectives); and used a range of frameworks, including cost utility and cost consequence analysis, to capture changes in the intersectoral costs and outcomes associated with child maltreatment.^52^ ^53^ The use of multiple economic evaluation frameworks provides decision makers with a comprehensive, multi-perspective guide to the cost effectiveness of the New Orleans Intervention Model.

### Phases

#### Developing or identifying a complex intervention

Development refers to the whole process of designing and planning an intervention, from initial conception through to feasibility, pilot, or evaluation study. Guidance on intervention development has recently been developed through the INDEX study^4^; although here we highlight that complex intervention research does not always begin with new or researcher led interventions. For example:

- A key source of intervention development might be an intervention that has been developed elsewhere and has the possibility of being adapted to a new context. Adaptation of existing interventions could include adapting to a new population, to a new setting,^54^ ^55^ or to target other outcomes (eg, a smoking prevention intervention being adapted to tackle substance misuse and sexual health).^20^ ^56^ ^57^ A well developed programme theory can help identify what features of the antecedent intervention(s) need to be adapted for different applications, and the key mechanisms that should be retained even if delivered slightly differently.^54^ ^58^
- Policy or practice led interventions are an important focus of evaluation research. Again, uncovering the implicit theoretical basis of an intervention and developing a programme theory is essential to identifying key uncertainties and working out how the intervention might be evaluated. This step is important, even if rollout has begun, because it supports the identification of mechanisms of change, important contextual factors, and relevant outcome measures. For example, researchers evaluating the UK soft drinks industry levy developed a bounded conceptual system map to articulate their understanding (drawing on stakeholder views and document review) of how the intervention was expected to work. This system map guided the evaluation design and helped identify data sources to support evaluation.^45^ Another example is a recent analysis of the implicit theory of the NHS diabetes prevention programme, involving analysis of documentation by NHS England and four providers, showing that there was no explicit theoretical basis for the programme, and no logic model showing how the intervention was expected to work. This meant that the justification for the inclusion of intervention components was unclear.^59^

Intervention identification and intervention development represent two distinct pathways of evidence generation,^60^ but in both cases, the key considerations in this phase relate to the core elements described above.

#### Feasibility

A feasibility study should be designed to assess predefined progression criteria that relate to the evaluation design (eg, reducing uncertainty around recruitment, data collection, retention, outcomes, and analysis) or the intervention itself (eg, around optimal content and delivery, acceptability, adherence, likelihood of cost effectiveness, or capacity of providers to deliver the intervention). If the programme theory suggests that contextual or implementation factors might influence the acceptability, effectiveness, or cost effectiveness of the intervention, these questions should be considered.

Despite being overlooked or rushed in the past, the value of feasibility testing is now widely accepted with key terms and concepts well defined.^61^ ^62^ Before initiating a feasibility study, researchers should consider conducting an evaluability assessment to determine whether and how an intervention can usefully be evaluated. Evaluability assessment involves collaboration with stakeholders to reach agreement on the expected outcomes of the intervention, the data that could be collected to assess processes and outcomes, and the options for designing the evaluation.^63^ The end result is a recommendation on whether an evaluation is feasible, whether it can be carried out at a reasonable cost, and by which methods.^64^

Economic modelling can be undertaken at the feasibility stage to assess the likelihood that the expected benefits of the intervention justify the costs (including the cost of further research), and to help decision makers decide whether proceeding to a full scale evaluation is worthwhile.^65^ Depending on the results of the feasibility study, further work might be required to progressively refine the intervention before embarking on a full scale evaluation.

#### Evaluation

The new framework defines evaluation as going beyond asking whether an intervention works (in the sense of achieving its intended outcome), to a broader range of questions including identifying what other impact it has, theorising how it works, taking account of how it interacts with the context in which it is implemented, how it contributes to system change, and how the evidence can be used to support decision making in the real world. This implies a shift from an exclusive focus on obtaining unbiased estimates of effectiveness^66^ towards prioritising the usefulness of information for decision making in selecting the optimal research perspective and in prioritising answerable research questions.

A crucial aspect of evaluation design is the choice of outcome measures or evidence of change. Evaluators should work with stakeholders to assess which outcomes are most important, and how to deal with multiple outcomes in the analysis with due consideration of statistical power and transparent reporting. A sharp distinction between one primary outcome and several secondary outcomes is not necessarily appropriate, particularly where the programme theory identifies impacts across a range of domains. Where needed to support the research questions, prespecified subgroup analyses should be carried out and reported. Even where such analyses are underpowered, they should be included in the protocol because they might be useful for subsequent meta-analyses, or for developing hypotheses for testing in further research. Outcome measures could capture changes to a system rather than changes in individuals. Examples include changes in relationships within an organisation, the introduction of policies, changes in social norms, or normalisation of practice. Such system level outcomes include how changing the dynamics of one part of a system alters behaviours in other parts, such as the potential for displacement of smoking into the home after a public smoking ban.

A helpful illustration of the use of system level outcomes is the evaluation of the Delaware Young Health Program—an initiative to improve the health and wellbeing of young people in Delaware, USA. The intervention aimed to change underlying system dynamics, structures, and conditions, so the evaluation identified systems oriented research questions and methods. Three systems science methods were used: group model building and viable systems model assessment to identify underlying patterns and structures; and social network analysis to evaluate change in relationships over time.^67^

Researchers have many study designs to choose from, and different designs are optimally suited to consider different research questions and different circumstances.^68^ Extensions to standard designs of randomised controlled trials (including adaptive designs, SMART trials (sequential multiple assignment randomised trials), n-of-1 trials, and hybrid effectiveness-implementation designs) are important areas of methods development to improve the efficiency of complex intervention research.^69^ ^70^ ^71^ ^72^ Non-randomised designs and modelling approaches might work best if a randomised design is not practical, for example, in natural experiments or systems evaluations.^5^ ^73^ ^74^ A purely quantitative approach, using an experimental design with no additional elements such as a process evaluation, is rarely adequate for complex intervention research, where qualitative and mixed methods designs might be necessary to answer questions beyond effectiveness. In many evaluations, the nature of the intervention, the programme theory, or the priorities of stakeholders could lead to a greater focus on improving theories about how to intervene. In this view, effect estimates are inherently context bound, so that average effects are not a useful guide to decision makers working in different contexts. Contextualised understandings of how an intervention induces change might be more useful, as well as details on the most important enablers and constraints on its delivery across a range of settings.^7^

Process evaluation can answer questions around fidelity and quality of implementation (eg, what is implemented and how?), mechanisms of change (eg, how does the delivered intervention produce change?), and context (eg, how does context affect implementation and outcomes?).^7^ Process evaluation can help determine why an intervention fails unexpectedly or has unanticipated consequences, or why it works and how it can be optimised. Such findings can facilitate further development of the intervention programme theory.^75^ In a theory based or systems evaluation, there is not necessarily such a clear distinction between process and outcome evaluation as there is in an effectiveness study.^76^ These perspectives could prioritise theory building over evidence production and use case study or simulation methods to understand how outcomes or system behaviour are generated through intervention.^74^ ^77^

#### Implementation

Early consideration of implementation increases the potential of developing an intervention that can be widely adopted and maintained in real world settings. Implementation questions should be anticipated in the intervention programme theory, and considered throughout the phases of intervention development, feasibility testing, process, and outcome evaluation. Alongside implementation specific outcomes (such as reach or uptake of services), attention to the components of the implementation strategy, and contextual factors that support or hinder the achievement of impacts, are key. Some flexibility in intervention implementation might support intervention transferability into different contexts (an important aspect of long term implementation^78^), provided that the key functions of the programme are maintained, and that the adaptations made are clearly understood.^8^

In the ASSIST study,^20^ a school based, peer led intervention for smoking prevention, researchers considered implementation at each phase. The intervention was developed to have minimal disruption on school resources; the feasibility study resulted in intervention refinements to improve acceptability and improve reach to male students; and in the evaluation (cluster randomised controlled trial), the intervention was delivered as closely as possible to real world implementation. Drawing on the process evaluation, the implementation included an intervention manual that identified critical components and other components that could be adapted or dropped to allow flexible implementation while achieving delivery of the key mechanisms of change; and a training manual for the trainers and ongoing quality assurance built into rollout for the longer term.

In a natural experimental study, evaluation takes place during or after the implementation of the intervention in a real world context. Highly pragmatic effectiveness trials or specific hybrid effectiveness-implementation designs also combine effectiveness and implementation outcomes in one study, with the aim of reducing time for translation of research on effectiveness into routine practice.^72^ ^79^ ^80^

Implementation questions should be included in economic considerations during the early stages of intervention and study development. How the results of economic analyses are reported and presented to decision makers can affect whether and how they act on the results.^81^ A key consideration is how to deal with interventions across different sectors, where those paying for interventions and those receiving the benefits of them could differ, reducing the incentive to implement an intervention, even if shown to be beneficial and cost effective. Early engagement with appropriate stakeholders will help frame appropriate research questions and could anticipate any implementation challenges that might arise.^82^

## Conclusions

One of the motivations for developing this new framework was to answer calls for a change in research priorities, towards allocating greater effort and funding to research that can have the optimum impact on healthcare or population health outcomes. The framework challenges the view that unbiased estimates of effectiveness are the cardinal goal of evaluation. It asserts that improving theories and understanding how interventions contribute to change, including how they interact with their context and wider dynamic systems, is an equally important goal. For some complex intervention research problems, an efficacy or effectiveness perspective will be the optimal approach, and a randomised controlled trial will provide the best design to achieve an unbiased estimate. For others, alternative perspectives and designs might work better, or might be the only way to generate new knowledge to reduce decision maker uncertainty.

What is important for the future is that the scope of intervention research is not constrained by an unduly limited set of perspectives and approaches that might be less risky to commission and more likely to produce a clear and unbiased answer to a specific question. A bolder approach is needed—to include methods and perspectives where experience is still quite limited, but where we, supported by our workshop participants and respondents to our consultations, believe there is an urgent need to make progress. This endeavour will involve mainstreaming new methods that are not yet widely used, as well as undertaking methodological innovation and development. The deliberative and flexible approach that we encourage is intended to reduce research waste,^83^ maximise usefulness for decision makers, and increase the efficiency with which complex intervention research generates knowledge that contributes to health improvement.

Monitoring the use of the framework and evaluating its acceptability and impact is important but has been lacking in the past. We encourage research funders and journal editors to support the diversity of research perspectives and methods that are advocated here and to seek evidence that the core elements are attended to in research design and conduct. We have developed a checklist to support the preparation of funding applications, research protocols, and journal publications.^9^ This checklist offers one way to monitor impact of the guidance on researchers, funders, and journal editors.

We recommend that the guidance is continually updated, and future updates continue to adopt a broad, pluralist perspective. Given its wider scope, and the range of detailed guidance that is now available on specific methods and topics, we believe that the framework is best seen as meta-guidance. Further editions should be published in a fluid, web based format, and more frequently updated to incorporate new material, further case studies, and additional links to other new resources.

## Notes

Contributors: All authors made a substantial contribution to all stages of the development of the framework—they contributed to its development, drafting, and final approval. KS and LMa led the writing of the framework, and KS wrote the first draft of this paper. PC, SAS, and LMo provided critical insights to the development of the framework and contributed to writing both the framework and this paper. KS, LMa, SAS, PC, and LMo facilitated the expert workshop, KS and LMa developed the gap analysis and led the analysis of the consultation. KAB, NC, and EM contributed the economic components to the framework. The scientific advisory group (JB, JMB, DPF, MP, JR-M, and MW) provided feedback and edits on drafts of the framework, with particular attention to process evaluation (JB), clinical research (JMB), implementation (JR-M, DPF), systems perspective (MP), theory based perspective (JR-M), and population health (MW). LMo is senior author. KS and LMo are the guarantors of this work and accept the full responsibility for the finished article. The corresponding author attests that all listed authors meet authorship criteria and that no others meeting authorship criteria have been omitted.

Patient and public involvement: This project was methodological; views of patients and the public were included at the open consultation stage of the update. The open consultation, involving access to an initial draft, was promoted to our networks via email and digital channels, such as our unit Twitter account (@theSPHSU). We received five responses from people who identified as service users (rather than researchers or professionals in a relevant capacity). Their input included helpful feedback on the main complexity diagram, the different research perspectives, the challenge of moving interventions between different contexts and overall readability and accessibility of the document. Several respondents also highlighted useful signposts to include for readers. Various dissemination events are planned, but as this project is methodological we will not specifically disseminate to patients and the public beyond the planned dissemination activities.

Provenance and peer review: Not commissioned; externally peer reviewed.
